# Emergent topological fields and relativistic phonons within the thermoelectricity in topological insulators

**DOI:** 10.1038/s41598-021-93667-x

**Published:** 2021-07-12

**Authors:** Daniel Faílde, Daniel Baldomir

**Affiliations:** Departamento de Física Aplicada, Instituto de Investigacións Tecnolóxicas, Universidade de Santiago de Compostela, Campus Vida s/n, 15782 Santiago de Compostela, Spain

**Keywords:** Topological insulators, Surfaces, interfaces and thin films, Thermoelectrics

## Abstract

Topological edge states are predicted to be responsible for the high efficient thermoelectric response of topological insulators, currently the best thermoelectric materials. However, to explain their figure of merit the coexistence of topological electrons, entropy and phonons can not be considered independently. In a background that puts together electrodynamics and topology, through an expression for the topological intrinsic field, we treat relativistic phonons within the topological surface showing their ability to modulate the Berry curvature of the bands and then playing a fundamental role in the thermoelectric effect. Finally, we show how the topological insulators under such relativistic thermal excitations keep time reversal symmetry allowing the observation of high figures of merit at high temperatures. The emergence of this new intrinsic topological field and other constraints are suitable to have experimental consequences opening new possibilities of improving the efficiency of this topological effect for their based technology.

## Introduction

The purely experimental fact that nowadays the best room temperature thermoelectrics are topological insulators (TIs)^1^, not only deserves a deep explanation to satisfy our scientific curiosity, but it also could greatly help in the development of new renewable energy technologies. The efficiency of heat transformation into electricity (or vice versa) in a thermoelectric material is measured with a parameter called the dimensionless figure of merit *ZT*, where *Z* is the figure of merit and *T* the absolute temperature. Its highest value is around 2.4 at 300 K for Bi$_2$Te$_3$/Sb$_2$Te$_3$ superlattices^2^. These structures are built with topological insulators for decreasing the thermal conductivity as much as possible and take advance of the highly conducting edge channels present in TIs to provide a high efficient thermoelectric response^3,4^. Nevertheless, the technological applications still need a higher value to the previously mentioned^5^. Besides the experimental difficulties to obtain high-quality thin-films, eliminate bulk carrier transport or adjust the Fermi level to lie in the small topological gap there is not a physical model to guide us towards a new generation of thermoelectric devices by treating phonons or oscillations produced by temperature in the topological context. The problem is that physics is fundamentally local and topology must enter through those tools. With this purpose, we need to connect topology and electrodynamics in order to introduce relativistic phonons and thermal excitations together with the topological electrons at the surface. The scenario which arises after this study, supported on an obtained expression for the topological intrinsic field, solves most of the difficulties involved in the topological thermoelectricity and allows to find experimental work to confirm it.

The global characteristics of TIs are printed on their band structure within their first Brillouin zone (BZ), concretely through their curvatures and Berry’s phases. These materials are both insulators in the bulk and semimetals on their surface where they contain Dirac points on account of the band crossings. But the bands are not the unique factor to take into account to determine the topology. One fundamental ingredient is the dimensions of the spacetime where the Hamiltonian is defined. That is why generally, for a given Hamiltonian *H*(*k*) following Schrödinger equation, we are able to define a map from the BZ to the manifold of their symmetries $\nonumber X(k): BZ \longmapsto \frac{U(n)}{U(n-i)\times U(i)}$ by means of a unitary matrix *U*(*n*) which diagonalizes the Hamiltonian for *n* states (Bloch bands) being *i* the occupied ones and $n-i$ the unoccupied with respect to the Fermi level. This allows calculating the number of maps *X*(*k*) which cannot be deformed continuously each other using the homotopy group obtaining $\pi _1[X(k)]=\pi _3[X(k)]=0$ and $\pi _2[X(k)]={\mathbb {Z}}$, being ${\mathbb {Z}}$ an integer and each subindex the dimension of the spacetime^6^. That means that there is only one non-trivial topology associated with the direct relabeling of the bands in two dimensions. Or in other words, band insulators with different integers cannot be continuously deformed into each other without crossing a quantum phase transition. This is what happens, for instance, in the Integer Quantum Hall Effect, where the number of chiral edge states are the integers associated to the above homotopy group^7–10^. But in one or three dimensions we need to introduce more symmetries of the Hamiltonian if we want to take into account their non-trivial topology. In TIs this symmetry is the time-reversal symmetry ${\hat{T}}$ which induces a Kramer’s degeneracy^11,12^, such that the square of ${\hat{T}}$ operator is equal to $-1$ for half-spin electrons (fermions), acting on the map previously defined as ${\hat{T}}X(k){\hat{T}}^{-1}=X(-k)$. This enables us to define a new topological number by the discrete cyclic group of two elements $Z_2$ called spin-Chern number^11,13^. The form to do it is somewhat subtle dividing the Hilbert space into two parts, one for each kind of Kramers states, and calculating the Chern number on them for extending the definition of the topological index^14,15^.

Once we know how the non-trivial topology can be determined on the bands, we need to figure up how the electromagnetic fields and heat exchange behaves physically under such non-trivial topologies. For such aim, we need to employ the axion electrodynamics which enlarges the Maxwell’s action using the two Lorentz invariants associated to the fields. That is to say, to add the pseudo scalar quantity $\frac{1}{2}\epsilon _{\mu \nu \alpha \beta }F^{\mu \nu }F^{\alpha \beta }$, besides the usual scalar $F_{\alpha \beta }F^{\alpha \beta }$ which is enough to provide Maxwell electrodynamics in the trivial topological vacuum. Then, we have the axion action ${\mathcal {S}}=\int dx^4\left( \frac{1}{4\mu _0c}F_{\alpha \beta }F^{\alpha \beta }-\frac{e^2}{32\pi ^2\hbar }\theta (r,t)\epsilon _{\mu \nu \alpha \beta }F^{\mu \nu }F^{\alpha \beta }+\frac{1}{c}A_{\mu }J^{\mu }\right)$ which leads directly to the equivalent Maxwell equations in TIs, $\varvec{\nabla } \left( {\varvec{E}}+2{\alpha c} \left( \frac{\theta }{2\pi } \right) {\varvec{B}}\right) =\frac{\rho }{ \epsilon _0}$ substituting the Gauss law and $\varvec{\nabla } \times {\varvec{B}}=\mu _0{\varvec{J}}+\frac{1}{c^2}\frac{\partial {\varvec{E}}}{\partial t}+\frac{2\alpha }{c} \left[ {\varvec{B}}\frac{\partial }{\partial t}\left( \frac{\theta }{2\pi }\right) +\varvec{\nabla }\left( \frac{\theta }{2\pi }\right) \times {\varvec{E}} \right]$ instead of the Maxwell-Ampere one, being $\alpha = \frac{e^2}{4\pi \epsilon _0\hbar c}$ the dimensionless fine structure constant. The other two equations, Faraday and non-existence of isolated magnetic poles, maintain the same form^16–18^. In particular for the TIs, $\theta =\pi$ and we can choose a gauge function $\Lambda (r,t)=\frac{\hbar 2\pi r}{ea}$ associated to the degree of freedom of the electromagnetic potentials where *a* is a lattice constant and |*r*| the modulus of the distance given in the electromagnetic fields. By constraining these fields on a torus, these ingredients are enough to connect $\theta$ with the non-trivial topologies of the lattice using Gauss–Bonnet, however, not with the bands through the Chern number. To reach such result we are going to translate the topological information into a physical field named as *b*. This will be done through the Berry curvature $\Omega _{k_x,k_y}=-2Im {\langle {\partial _{k_x}n}|}{{\partial _{k_y}n}\rangle }$ defined on the non-trivial bands of a 2D Dirac Hamiltonian which behaves as a spin-dependent magnetic field in the *k*-space and whose integral determines the Chern number *C*.

**Figure 1:**
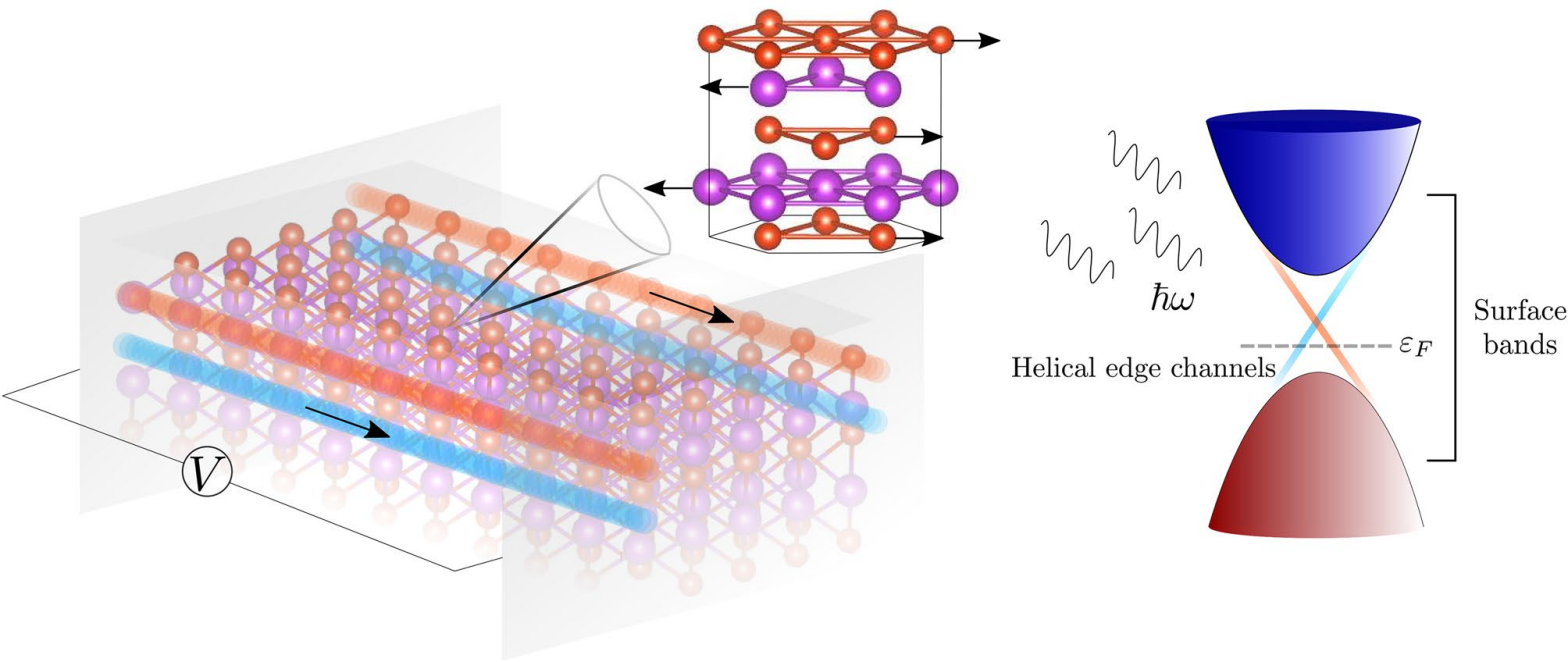
Lattice oscillations in the topological electronic transport. Schematic illustration of helical edge states and surface of a 3DTI in thin-film conditions. The energy spectrum is displayed on the right side of the panel. The quintuple layer describes the addition of in-plane lattice oscillations, in this case represented by a polar phonon mode, to the topological electronic transport.

Supported by this topological field, which is intrinsic to these materials and consistent with their special electromagnetic background, we are able to introduce and interpret the thermodynamic part associated to the phonons in TIs (Fig. 1). This is done by including oscillations in the Dirac Hamiltonian, i.e., relativistic phonons associated to the Dirac oscillator^19^. By means of the adiabatic mechanism, we are going to establish an equivalence between the phonon field $\varvec{\omega }$ of the Dirac oscillator and the field ${\varvec{b}}$ containing the information of the dynamic and the robustness of the topological regime. This is a crucial result for the topological formalism of the thermoelectricity. On the one hand, we demonstrate in a direct way how relativistic phonons enters into the topological context, modifying the Berry curvature and allowing heat-electricity transformation. On the other hand, we give an explanation of why topology is preserved at high temperatures in most of the compounds which exhibit it. The previously mentioned relationship between the field ${\varvec{b}}$, which can reach values in the order of Teslas for each spin subsystem with the typical parameters in 3DTI thin films, and $\omega$ defines a limit (> THz) for the frequencies tolerated by the system without involving entropy change. Under these conditions, we would find a temperature regime in which such excitations would not break the quantum coherence necessary for the conservation of the topological signatures allowing the observation of the high figures of merit associated to the topological states around room temperature.

## Results

The family of 3DTIs Bi$_2$Se$_3$, Bi$_2$Te$_3$, Sb$_2$Te$_3$ has a special interest by the fact of being topological besides including on their members the most efficient thermoelectric material up to now. The highly conducting edge states, provided by the topology, are predicted to be responsible for their high figure of merit in low thermal conductivity conditions^4,20^. However, this must not be the unique ingredient to explain the thermoelectricity in these materials where the coexistence of time-reversal symmetry and non-zero temperatures, which usually involves entropy change, might cause a conflict. Given that, our starting point must be a 2D effective Dirac Hamiltonian used to describe the physics inside 2DTIs as well as in 3DTIs thin films, i.e., when the thickness of a 3DTI is enough small to overlap its top and bottom surface states forcing them to be placed at the edge^21–23^.

1 $$\begin{aligned} H_{2D}(\mathbf{k})=\left[ \begin{array}{ll} {H_+} &{} {0} \\ 0 &{} H_- \\ \end{array} \right] , \quad H_{\pm } = \left( \begin{array}{ll} \pm M(\mathbf{k}) &{} \hbar v_F k_-\\ \hbar v_F k_+ &{} \mp M(\mathbf{k}) \end{array}\right) \end{aligned}$$

**Figure 2:**
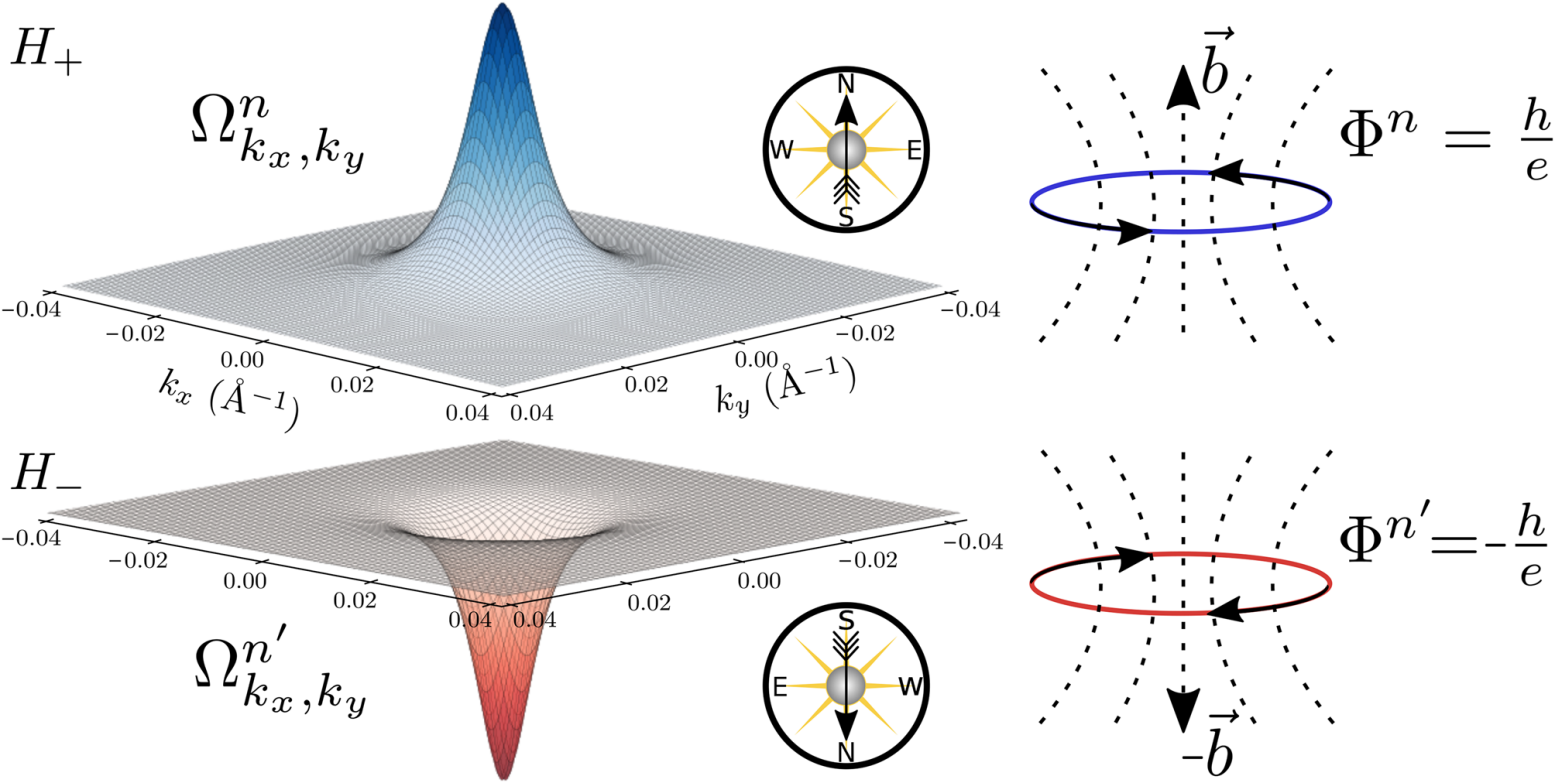
Non-trivial Berry curvature and effective flux quantization in TIs. Non-trivial ($M<0$, $B<0$) Berry curvature $\varvec{\Omega }_{k_x,k_y}$ for the positive energy eigenstates of $H_\pm$ labelled as ${|{n}\rangle }$ and ${|{n'}\rangle }$ respectively. The compass indicates the orientation of the field felt by the electrons on each band. In the bulk of a TI, this curvature allows considering the existence of helical orbits with an associated flux $\Phi =h /e \; C$, being $C=\pm 1$ the Chern number associated to the conduction bands of $H_\pm$.

Here $k_\pm =k_x\pm ik_y$, $M({\mathbf{k}})=M-{\mathcal {B}}k^2$ is the effective mass in the solid, $k^2=k_x^2+k_y^2$, $v_F$ is the Fermi velocity, $\hbar$ is the Planck’s bar constant and the basis has been rearranged to be $\left[ \psi _{1\uparrow }, \psi _{2\downarrow }, \psi _{2\uparrow }, \psi _{1\downarrow } \right]$, allowing the separation of the Hamiltonian into two non-interacting time-reversal counterparts $H_\pm$ which can be treated independently^22,24^. The energy spectrum of Eq. (1) define two non-interacting Dirac hyperbolas centered at the $\Gamma$ point for which conduction and valence bands for $H_+$ have an associated Berry curvature

2 $$\begin{aligned} \varvec{\Omega }^{c}_{k_xk_y}=-\varvec{\Omega }^{v}_{k_xk_y}=-\frac{\hbar ^2v_F^2(M+{\mathcal {B}}k^2)}{2[(M-{\mathcal {B}}k^2)^2+\hbar ^2v_F^2k^2]^{3/2}} \varvec{{\hat{z}}} \end{aligned}$$

which is spin and band dependent, resulting in the opposite sign for $H_-$ ($M\rightarrow -M$). This Berry curvature defines the Chern number $C=1/(2\pi ) \int \varvec{\Omega }d {\varvec{k}}$ which in the non-trivial regime of $H_{2D}$, given by the condition $M{\mathcal {B}}>0$, is an integer equal to $\pm 1$ which also shares the same dependence of $\varvec{\Omega }$ and its responsible of transport quantization^22^. Essentially, the Berry curvature plays the role of a magnetic field in the k-space obtained through the rotational of the Berry potential ${\varvec{A}}$. This allows us to consider in a TI, the presence of a pair spin-momentum locking orbits associated to the topology, which present a quantized flux ($C \; h/e$) in terms of the Chern number *C* and whose sum obviously gives zero due to ${\hat{T}}$ symmetry (Fig. 2). Of course, this is consistent with the fact of why in the presence of an in-plane electric field we can talk about opposite transverse spin currents which in the edge produce a quantized electrical conductance $G=(C_+-C_-) e^2/h$, being $C_\pm$ the Chern numbers associated with the branches $H_\pm$^25,26^. The idea to understand and address effects associated with thermal fields and phonons or external fields is to link the topological information into a physical field consistent with their phenomenology. But, how can it be done? The answer lies in the translation of the Berry curvature into the real space. This can be done by noticing that under small gap conditions, as it happens for 3DTIs in the thin-film limit, the non-trivial Berry curvature $\varvec{\Omega }_{k_x,k_y}$ associated to the states of Eq. (1) has the form of a single peak Gaussian-like function centered at the $\Gamma$ point^22^, which has characteristic length small enough to consider an equivalent magnetic field ${\varvec{b}}$ constant along the bulk crystal and whose magnitude must be determined by the constraint that its flux is quantized and equal to $h/e \; C$, i.e., $\hbar /e \int \varvec{\Omega }_{k_x,k_y} d{\varvec{k}} = \int {\varvec{b}} d{\varvec{S}}$.

Given that *b* can be extracted from the integral, we need now to estimate the area defined by the topological electrons on their motion. This surface element $\Delta {\varvec{S}}$ can be obtained in an original way by applying Heisenberg’s uncertainty principle matching the quantum conductance ($e^2/h$) with the conductivity $\sigma =\Delta S^{-1} \frac{e^2 \tau }{m_{e}}$ in the Heisenberg limit ($\tau =\hbar /\Delta \varepsilon$) being *e* the elementary charge, $\tau$ the scattering time and $\Delta \varepsilon =2\xi$ the energy uncertainty which can be considered to be in the order of the energy of the particle $\xi$ due to the low energy of the topological electrons and that we take equal to $2\xi$, i.e., the energy difference between the two eigenstates^27^. In this way, we obtain the following expression for the field ${\varvec{b}}$

3 $$\begin{aligned} {\mathbf{b}} =\frac{2 m_e \xi }{\hbar e} C \; \hat{\mathbf{z }} \approx \frac{2m_e^2v_F^2}{\hbar e} C \; \hat{\mathbf{z }} \end{aligned}$$

which can be approximated by setting $\xi \approx m_e v_F^2$ in account of the small momentum of the particles and where the electron effective mass can be considered as $m_{e}=\hbar ^2(\frac{\partial ^2\xi }{\partial k^2})^{-1}=M/v_F^2$ neglecting any contribution from the Hamiltonian parameter ${\mathcal {B}}$, that gives us the information about the localization or delocalization of the bands in the space, and limiting to materials that present $v_F^2>>2M{\mathcal {B}}/\hbar ^2$. This approximation, easily fulfilled thanks to the small gap and high Fermi velocity that typically characterizes 3DTI thin films ($M\approx -25$ meV, $v_F=6.17 \; 10^5$ m/s), determines an equivalent field for electrons on the surface $\left|b\right|\approx 5$ T for these values consistent with the robustness that characterizes topological surface states and whose sign, determined by the Chern number, keeps time-reversal symmetry intact. This expression for *b* is not far from the one obtained directly by transforming a narrow Gaussian function from the momentum space to the real space where its value is determined by the inverse of the maximum of the source function. In this way, the large magnitude of *b* is uniquely due to the size of Berry curvature which is associated to the band singularities. Note that in Eq. (3) $m_e$ must not change its sign when we pass from $H_+$ to $H_-$ ($C\rightarrow - C$) given that its inversion has already been considered in the conductivity change $e^2/h\rightarrow -e^2/h$. Thus, our formalism defines an effective area $\Delta S=h\hbar /(2m_e \xi )$ for the electrons inside TIs, which corresponds with the inverse of the 2D density of states, and a magnetic field *b* with opposite sign for each band and branch $H_\pm$, which attending to the full Hamiltonian, results in a special spin-dependent interaction consistent with the singular dynamic of the topological regime and the band inversion. The obtained expression for the field *b* also shares a direct correspondence with the critical magnetic field $B_c=m^2c^2/(\hbar e)$ necessary for two photons to create Schwinger pairs in the vacuum^28^, adapted to the particular context of the TIs due to the small gap and the substitution of *c* by $v_F$, making this quantity experimentally accessible. Additionally, it can be checked that indeed Eq. (3) matches with the critical value of the external magnetic field *B* making zero the density of states and Fermi sea volume in non-zero Berry curvature systems at $k=0$, being these quantities proportional to the factor $(1+\frac{e{\varvec{B}}\cdot \varvec{\Omega }}{\hbar })$^29,30^.

**Figure 3:**
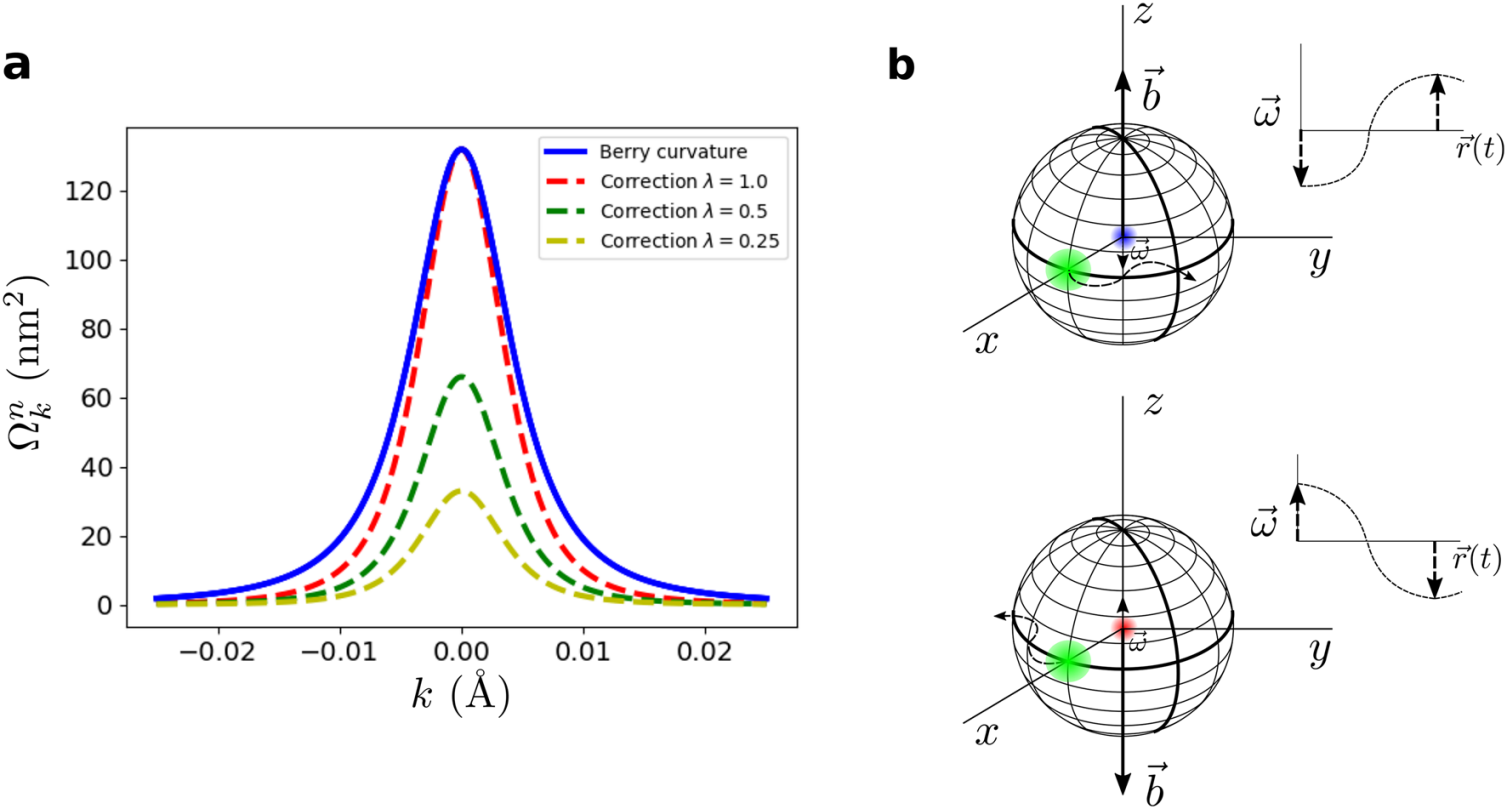
Berry curvature correction and b field interpretation. (**a**) Unperturbed Berry curvature (blue solid line) of the conduction band ($H_+$) of a topological insulator ($M<0$) and first-order correction to it (dashed lines) for different energy dependent frequencies below the critical frequency $\omega _c=eb/2m$. The parameters used are $M=-0.025$ eV, $\mathcal{B}=0$ and $v_F=6.17 \times 10^5$ m/s. (**b**) Schematic translation of Berry curvature modulation from the electronic point of view (blue and red points) where in-plane nucleus (green circles) displacements are showed to change the field *b* defined on each orbital motion. Red and blue points represent electrons with opposite spins for which nucleus move in opposite directions attending to the helical nature of electronic motion in a TI.

As we are going to see, now we are in a position to interpret the role of relativistic oscillations in TIs. It is straightforward to show that the abstract remarks defined above are closely related to the phenomenology of the Dirac oscillator Hamiltonian $i\hbar (\partial \psi / \partial t)=[v_F \varvec{\alpha }({\varvec{p}}-im {\varvec{r}}\omega \beta )+mv_F^2\beta ] \psi$, which also preserves time-reversal symmetry and incorporates a linear correction in *r* to the electron momentum *p* in the form of a magnetic field $B=2m\omega /e$. In a 2D Hamiltonian as Eq. (1) the perturbation introduced in the Dirac oscillator enters in the same way of a magnetic field *B* in the *z*-direction with opposite sign for each branch $H_\pm$ with the usual substitution $2\omega =eB/m$ with a difference of a factor 2 that comes from the non-minimal coupling present in the Dirac oscillator equation to guarantee of having a harmonic oscillator in its non-relativistic limit^19,31^. Precisely in this limit, is where a spin-orbit coupling of strength $2\omega /\hbar$ arises motivated by the spin-dependent magnetic interaction introduced in the system. The Dirac oscillator (“Supplementary Information”) has been analyzed in different studies^19,31–33^, however, it has not been treated into the adiabatic formalism. Only then, we are going to be capable of visualizing the role of phonons inside the topological context. Given that the Hilbert space was divided into two time-reversal counterparts, we are able to work in only one of the subsystems $H_\pm$ where the adiabatic correction to the energy eigenstates can be calculated^34,35^. The Lorentz force provided by $\varvec{\omega }$ increases or decreases (depending on its sign) the electron momentum in the direction *i* proportionally to their perpendicular components. In that way, as a first approximation to the problem we can formulate the temporal variation of the momentum as $\partial k_i / \partial t=\epsilon _{ijk} k_j \omega _k$ and, regrouping terms, write the correction to the eigenstates given by the Dirac oscillator

4 $$\begin{aligned} {|{n}\rangle } \rightarrow {|{n}\rangle } + \frac{\hbar \omega }{4\xi ^2} \hbar v_F k {|{m}\rangle } \end{aligned}$$

where ${|{n}\rangle }$ and ${|{m}\rangle }$ are the positive and negative energy eigenstates of $H_+$. From here, we compute after some tedious algebra the corrections to the Berry curvature of the bands $\Omega ^{i}_{k_xk_y}=-2Im {\langle {\partial _{k_x} i}|}{|{\partial _{k_y}i}\rangle }$. Two cases were analyzed, both sharing an ability to modulate the Berry curvature (“Supplementary Information”). The first one, considering a purely uniform $\omega$, represents the phonons or oscillations with a constant energy dispersion and their corrections have the form of a function similar to the Berry curvature which changes its sign at some point *k* (Supplementary Fig. S1). Besides being quite restrictive, these modes will not be suitable to introduce in the thermoelectric mechanism. The second case considers an explicit energy dependence on the phonon frequency, i.e. $\hbar \omega =\uplambda \xi$, which not only could maximize the coupling with the topological electrons given its relativistic energy dispersion but at the same time is consistent with its apparent relation with the intrinsic topological field $b=2m\omega /e$, with no more ingredients that substituting Eq. (3) into the previous relation. Thus, made the calculations, curvature corrections turn out to be

5 $$\begin{aligned} \Omega ^n(k) \rightarrow \Omega ^n(k) - \uplambda \frac{M}{\xi } \Omega ^n(k) \end{aligned}$$

where $\uplambda$ ($\in [0,1]$) is a dimensionless parameter measuring the relative strength of $\omega$ with respect to *b*. The obtained results, plotted in Fig. 3, demonstrates how phonons and oscillations can be introduced into the context of TIs modulating the Berry curvature and hence the field *b* even when the perturbation is not small compared with the energy of the system but whose variation can be adiabatic. For the limit case $\uplambda =1$, i.e. $\omega =eb/2m$, the obtained first-order correction have the same height at $\Gamma =0$ than the unperturbed Berry curvature. This confirms the correctness of the expression for the field *b* and its interpretation as a measure of the topological robustness and as a critical value for the strength of in-plane oscillations and external forces (strain, spin-orbit) supported by the surface states. Beyond this limit, the Berry curvature will change its sign. Of course, these effects are not produced by every phonon mode presented in the crystal but some specific phonons for which the concept helicity must be involved in order to couple topological electrons and capable to cause a correction to the momentum of $\partial k_i / \partial t=\epsilon _{ijk} k_j \omega _k$, i.e., relativistic. This constraint, that will need a suitable phonon dispersion, has been shown to be on the energy range needed for polar optical modes in Bi$_2$Te$_3$ and Bi$_2$Se$_3$^36^. The expressions derived above are also valid in the case of taking into account the dependence of the mass term $M(k)=M-{\mathcal {B}}k^2$ on the Hamiltonian parameter ${\mathcal {B}}$ (Supplementary Fig. S2). In all cases, as it is expected, it can be shown that the corrections introduced respects the intrinsic particle-hole ${\hat{C}}$ and ${\hat{T}}$ symmetries of $H_{2D}$. Finally, it should be noted that the curvature corrections displayed in Eq. (5) are valid for quantitative purposes and no for a faithful determination of the Chern number by means of their integral. A full perturbational derivation of these corrections implies that the temporal variation of the particle momentum must be written in terms of the velocity operator and no with the momentum^29^. However, the approximation used is suitable as an estimation of the effects produced by the perturbations simplifying the calculations and avoiding to deal with gauge dependent corrections.

Both, electrodynamics and bands, have a common degree of freedom given by the gauge transformations within the Abelian group U(1). Mathematically they also share non-trivial topological features where the homotopy group $\pi _1(U(1))$ is equivalent to the integer numbers: the bands through the Berry curvature and the electrodynamics through torus constraints with genus equal one. Let us to develop it deeper from the physical consequences. Using the periodicity of the Brillouin zones, we can define the function $\Lambda (r,t)=\frac{\hbar 2\pi r}{ea}$, where a is the lattice constant, which allows a transformation of the potentials such that $A_{\mu }\longrightarrow A'_{\mu } =A_{\mu }+\partial _{\mu } \Lambda (r,t)$. This makes the space-time to be on a torus of four dimensions $T^4=T^2 \times T^2$ for the electromagnetic fields and taking $\pi$ degrees between *E* and *B*, i.e. sharing a spatial direction. This leads to a quantized electric and magnetic fields, $B=n \frac{h}{e a^2}$ and $E=n' \frac{h c}{e a^2}$, where $n,n' \in {\mathcal {N}}$ are natural numbers which determine the topological sector. Coming back to the *EB* term of the action we can see how this part of the action is quantized making $\theta$ belong to the interval $[0,2\pi )$ and therefore obtaining a topological vacuum angle that usually is written as ${|{\theta }\rangle }=\sum _\nu exp(i \nu \theta ) {|{\nu }\rangle }$, being $\nu$ the winding number^18^. The defined electromagnetic background can be now easily connected with the topology of the bands through the intrinsic topological field *b*, substituting the area $a^2$ by the one $\Delta S=h\hbar /(2m_e \xi )$ obtained for the electrons in a TI, resulting that

6 $$\begin{aligned} B=\frac{2m_e\xi }{\hbar e}\; C\equiv b \end{aligned}$$

where *n* is now interpreted as the Chern number *C*. Notice that *b* is associated to the curvature of the bands, while *B* is a pure magnetic field of axion electrodynamics carrying information of the non-trivial topology on the spacetime. Both things are conceptually very different, one worked with Chern numbers and the other with genus using Gauss–Bonnet theorem; but physically they are connected linking the singularities of the bands and the real spacetime. Consistently, by making the same substitution we also find an expression for the electric field $E=2m_e^2v_F^3n'/\hbar e$ inside TIs, obtaining a value that determines the field needed to force a topological phase transition and which is again related with the corresponding critical field $E_c$ in the vacuum needed to create electron–hole pairs^4,37,38^. This is all that we can have for the electromagnetic fields within the TI with constant $\theta$, they are fixed and hence can not intervene in the dynamics, but we can overcome this difficulty just introducing phonons that move the different topological sectors. In this case the lattice constant depends of time and also the above electric and magnetic fields. Berry curvature also do it, as we have shown, keeping gauge invariant the bands with respect to the electromagnetic potentials.

**Figure 4:**
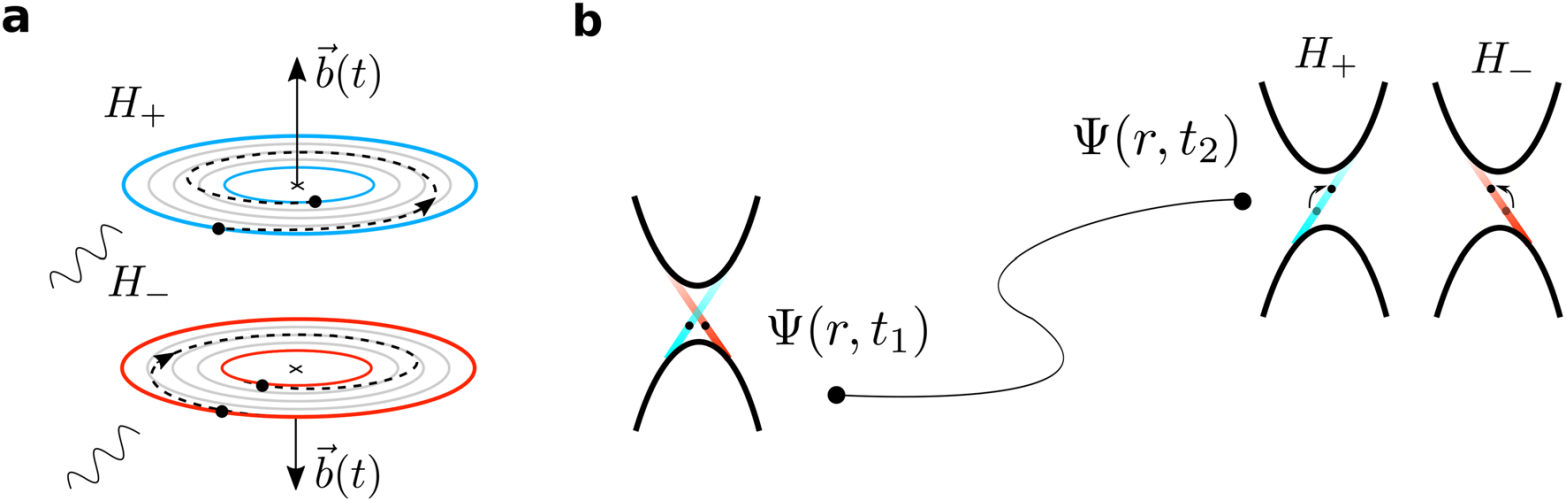
Adiabatic coherent process with $\partial C/\partial T=0$ (**a**) Illustration of an adiabatic coherent process, for which the magnitude of the field *b* increases. Blue and red circles represent the initial and resulting final states on each branch defined in $H_{2D}$. (**b**) Adiabatic coherent evolution of electronic wavefuntion $\Psi (r,t)$. Seebeck coefficient and entropy are not modified in this process allowing heat-electricity transformation in a completely reversible process. As a consequence of the enlargement of the *b* field and the Berry curvature, the moment of the possible final states $p_2$ are higher than the initial electron moment $p_1$. Below the critical value, edge states remain to be ballistic with their conductance quantized.

Therefore, we can tackle the phenomena of topological thermoelectricity by means of the uncertainty principle, equivalently to a treatment through the instanton solutions of the Chern–Simons action^4^, with an effective field *b* that varies due to the effects of phonons or thermal oscillations. In that case, the role of temperature into the topological transport was addressed by introducing it in the electromagnetic potentials through the gauge transformation $\Lambda ({\bar{\tau }})=\frac{2\pi n {\bar{\tau }}}{e\beta }$ being ${\bar{\tau }}=\hbar \beta =\hbar /k_B T$ the Euclidean time, but now can be joined up directly by assigning to the energy $\Delta \varepsilon$ an adiabatic temperature dependence $k_B T$. Note that there is an infinite number of orbits for which the relation between *b* and $\Delta S$ defines a quantized flux *h*/*e*. Thus, when a phonon or a thermal excitation couples to a topological electron, the electron must move towards another orbit consistent with its new *b*, curvature and energy in order to maintain the quantum flux $h/e \; C$ constant. In this way, given that all these states share the same electrical conductance $G=\Delta I/\Delta V=e^2/h$ in presence of an external electric field, we can directly find the contribution to the electric potential $\Delta V=\Delta I \, h/e^2$ generated by a thermal gradient, and vice versa, by substituting the electric current $\Delta I=e/\tau$ with the scattering time $\tau =\hbar /\Delta \varepsilon =\hbar /k_B T$ defined in Eq. (3). Thus, we can obtain the change in the electric potential due to thermal effects

7 $$\begin{aligned} V= V' + \frac{2\pi }{e} C k_B T \end{aligned}$$

and also its associated electric field

8 $$\begin{aligned} {\varvec{E}}= -\frac{2\pi }{e} C k_B \varvec{\nabla } T - \frac{2\pi }{e} \frac{\partial C}{\partial r} k_B T \; \hat{{\varvec{r}}} \end{aligned}$$

where the Chern number C takes into account the number of channels taking part in the thermalization of the system. The resulting expressions are identical to that obtained in Ref.^4^ introducing the thermodynamic part in TIs through a Chern Simons action. The second term in Eq. (8) makes reference for strong thermal perturbations which are able to change the Chern number, producing an anomalous Seebeck contribution $S=\frac{2\pi }{e} k_B \frac{\partial C}{\partial T}$ associated to the creation of electron-hole Schwinger pairs or to jumps between bands with different Chern numbers. In contrast, the first one take into account the perturbations which can produce changes in the Berry curvature without changing the Chern number. Focusing on this regime, the explanation of why we can observe the high thermoelectric response associated to the topological edge states around room temperature is immediate. Consider for instance a coherent process (Fig. 4a), in which a phonon or a thermal excitation couples to a topological electron increasing the field *b* and producing an electric field ${\varvec{E}}=-2\pi /e \; Ck_B \varvec{\nabla }T$. As it can be easily calculated, the Seebeck coefficient $S=\partial V/\partial T=2\pi /e \; k_B C$, and hence the entropy of the system, does not depend on temperature. That is, all the possible final states, associated to a small or a large adiabatic perturbation, share the same Seebeck coefficient, keeping the entropy constant, up to the limit in which the second term of Eq. (8) must be considered. In this way, the coherence needed to observe quantized topological signatures would still being conserved up to a temperature $T\sim 2mv_F^2/k_B$ proportional to the band gap of the system ($\Delta \varepsilon =k_BT$). Notice that the sum of all the entropy associated with these processes is zero, as the Chern numbers do when there is time-reversal symmetry. The translation of these results to edge physics is clear. The electric field generated due to the temporal variation of their intrinsic *b* turns into an enhancement (coherent) or reduction (decoherent) of the relative moment between the two helical currents, however, the edge states remain to be ballistic with a quantized conductance $G=e^2/h \; C$ up to the Chern changes to zero or the coherence is lost (Fig. 4b). These results stand uniquely for the thermoelectric response of the edge states without considering quantum interference effects between the top and bottom surfaces in the case of 3DTIs thin films and neglecting contributions from the bulk carriers^39^. From here, it can be obtained an expression for the topological figure of merit *ZT* associated with the edge states^4^. This is done through a topological formalism which gives a Seebeck coefficient, that despite having a different physical origin and interpretation is in the order of the ones resulting in the Landauer transport formalism from optimizing the position of the Fermi level $\xi _F$ in TIs with a large ratio between the edge and bulk scattering times^40^.

## Discussion

In summary, we present a formalism based on a purely effective topological intrinsic field *b* which allows to treat topology in a new way measuring its robustness at the same time it allows to incorporate relativistic phonons and thermal effects into the topological context. Below the limit defined, phonons and thermal excitations could couple to electrons without changing entropy, and hence, the Seebeck coefficient *S* and maintaining the coherence necessary to keep the quantum conductance $G=e^2/h \; C$ of its highly conducting channels invariant too. This fact is valid for a wide range of values given the magnitude of *b*, which for the parameters characterising 3DTI thin-films ($M\approx 0.025$ eV, $v_F=6 \times 10^5$ m/s) defines a field of 5 T and a frequency limit above the THz which can be generalizable to any topological insulators in 2D or for 3DTIs in thin film conditions with no more ingredients as their band gap, Fermi velocity and Chern number. This explains exactly how temperature and time-reversal symmetry coexist allowing the observation of the topological signatures and their associated high figure of merit at room temperature^23,41,42^. The ingredients provided are basic to derive the expression for the topological figure of merit ZT associated to the Kramers edge states in zero lattice thermal conductivity conditions^4^, but additionally, we define in which circumstances the current experimental limit can be overtaken^2^.

A new route to find high efficient thermoelectric devices by means of a special coupling between electrons and phonons at the surface is described^43^. In such situation there should be a depression in the number of phonon modes available in the system to contribute to thermal transport meaning a reduction in the lattice thermal conductivity and a heat-electricity transformation. This can be translated from an enhancement of electron-phonon coupling as it has been observed in 3DTIs Bi$_2$Se$_3$ and Bi$_2$Te$_3$ where polar optical phonon modes look to couple strongly with Dirac electrons when the Fermi level lies close to the Dirac point^36^. The map between these results and the model seems immediate since we are considering in-plane oscillations (polar) in our surface Hamiltonian at the same time as the enhancement of electron–phonon coupling takes place for frequencies satisfying the condition $2\omega =eb/m$ previously underlined. This could imply an alternative method to decrease lattice thermal conductivity for certain phonon modes as compared with other techniques (superlattices, impurities, dislocations) for increasing the thermoelectric efficiency or lead to exotic phenomena^2,44–46^. Additional experimental scenarios can be proposed to support our results. Besides a relativistic electron-phonon coupling and a high thermoelectric response, the study of the topological intrinsic field *b* can lead to more direct verification. Defined as an outcome of the topological robustness it enables us to define the critical magnetic and electric fields on each spin subsystem $H_\pm$ that could be measured.

## Supplementary information

Supplementary Information.

## Data Availability

The data that support the findings of this study are available from the corresponding authors upon reasonable request.
