# Supplementary material for: Emergent topological fields and relativistic phonons within the thermoelectricity in topological insulators
Source: Sci Rep. 2021 Jul 12;11:14335. doi: 10.1038/s41598-021-93667-x (PMC8275584; doi:10.1038/s41598-021-93667-x)
Supplement: Supplementary file 1 — Supplementary Information. [file 41598_2021_93667_MOESM1_ESM.pdf]

Supplementary Materials for

Emergent topological fields and relativistic phonons within the thermoelectricity in topological insulators

Daniel Failde,[1*] Daniel Baldomir[1*]

[1]Departamento de Física Aplicada, Instituto de Investigacións Tecnolóxicas,
Universidade de Santiago de Compostela,
E-15782 Campus Vida s/n, Santiago de Compostela, Spain

daniel.failde.balea@rai.usc.es, daniel.baldomir@usc.es

Topological insulators (TIs) are Quantum Spin Hall (QSH) systems with conductor states on their surface associated with a non-trivial topology of their electronic band structure [26]. On their edge, bands follow a linear dispersion law $E = \hbar v_F k$ as it would happen for a relativistic particle with zero rest mass which goes through singularity points (Dirac points). These singularities are the sources of the non-trivial topology, which, protected by time-reversal symmetry induce chiral Kramers currents that can be determined using Berry's gauge fields associated with their curvature on U(1) or SU(N) groups depending on band degeneracy [14, 15]. The robustness of these spin-momentum locking channels as well as their quantized transport properties, make three and two-dimensional TIs strong candidates in the context of Quantum Computing or Thermoelectric and Superconducting Devices. However, for a better understanding of these applications, some key points as thermal excitations and phonons have to be incorporated to electrons dynamic in TIs since their presence could lead to losing the quantum adiabaticity and coherence necessary to maintain such topological order [37].Along this line, we analyze the possible effect of phonons and thermal excitations in TIs surface states by studying adiabatic mechanical oscillations through Dirac oscillator model [19]. In order to see if non-trivial topology is preserved under certain values for the involved physical magnitudes in this process, we make use of the field interpretation of the Berry curvature, where magnetic flux quantization of helical orbits provides an argument to estimate the strength of the field b associated to the topological regime. With this background, we can find an equivalence between the oscillation frequency and the intrinsic field at the same time we analytically verify the robustness of the topological regime against phonons, thermal gradients and similar perturbations.

Supplementary Note 1. The Dirac oscillator

The introduction of mechanical oscillations in a relativistic context was first analyzed by M. Moshisnky and A. Szczepaniak [19] incorporating a linear term in r to the Dirac equation. The origin of this term lies in the introduction of an harmonic oscillator potential into the Klein-Gordon equation, leading to the well-known Dirac oscillator

$$i\hbar(\partial\psi/\partial t) = [v_F \boldsymbol{\alpha}(\boldsymbol{p} - im\boldsymbol{r}\omega\beta) + mv_F^2\beta]\psi \tag{1}$$

being $\alpha_i = \begin{bmatrix} 0 & \sigma_i \\ \sigma_i & 0 \end{bmatrix}$, $\beta = \begin{bmatrix} \sigma_0 & 0 \\ 0 & -\sigma_0 \end{bmatrix}$, σ_i the Pauli matrices, m the mass of the particle, r the position and where we have substituted the original speed of light c by the Fermi velocity v_F in order to adapt equation Eq. (1) into the context of TIs. Afterwards, the equation was further analyzed, always in the context of Quantum Field Theory (QFT) [30, 31], where working with phonons it is always convenient to employ operators defined on a Fock space and Eq. (1) can be rewritten in function of the right and left chiral annhilation and creation operators $a_r = \frac{1}{\sqrt{2}}(a_x - ia_y), a_r^+ = \frac{1}{\sqrt{2}}(a_x^+ + ia_y^+)$ and $a_l = \frac{1}{\sqrt{2}}(a_x + ia_y), a_l^+ = \frac{1}{\sqrt{2}}(a_x^+ - ia_y^+)$, being a_x, a_y, a_x^+ and a_y^+ the usual annihilation and creation operators of the harmonic oscillator. There are two Pauli spinor eigenstates which present entanglement between the spin and orbital degrees of freedom.

$$|\psi_1\rangle = i\frac{2mv_F^2\sqrt{\epsilon}}{E - mv_F^2} a_l^+ |\psi_2\rangle \tag{2}$$

$$|\psi_2\rangle = -i\frac{2mv_F^2\sqrt{\epsilon}}{E + mv_F^2} a_l^+ |\psi_1\rangle \tag{3}$$

being $|\psi_1\rangle$ and $|\psi_2\rangle$ the two components of the spinor $|\psi\rangle$, $\epsilon = \frac{\hbar\omega}{mv_F^2}$ takes into account the non-relativistic limit and $|n_l\rangle = \frac{1}{\sqrt{n_l!}}(a_l^+)^{n_l}|0\rangle$ the basis in which the Fock space is expanded [30]. The energy spectrum is $E = \pm E_{n_l} = \pm mv_F^2\sqrt{4\epsilon n_l + 1}$, whose eigenstates can be written as Pauli spinors $|\phi_\uparrow\rangle$ and $|\phi_\downarrow\rangle$ components, employing the angular momentum z-component definition given by $L_z = \hbar(a_r^+ a_r - a_l^+ a_l)$

$$|-E_{n_l}\rangle = \beta_{n_l}|n_l\rangle|\phi_\uparrow\rangle + i\alpha_{n_l}|n_l-1\rangle|\phi_\downarrow\rangle \tag{4}$$

$$|E_{n_l}\rangle = \alpha_{n_l}|n_l\rangle|\phi_\uparrow\rangle - i\beta_{n_l}|n_l-1\rangle|\phi_\downarrow\rangle \tag{5}$$

where $\alpha_{n_l} = \sqrt{\frac{E_{n_l}+mv_F^2}{2E_{n_l}}}$ and $\beta_{n_l} = \sqrt{\frac{E_{n_l}-mv_F^2}{2E_{n_l}}}$. Finally, time dependent state of the spinors excited by the Dirac oscillator is

$$|\psi(t)\rangle = \left(cos\ \omega_{n_l}t + \frac{i}{\sqrt{4\epsilon n_l+1}}sin\ \omega_{n_l}t\right)|n_l-1\rangle|\phi_\uparrow\rangle + \left(\sqrt{\frac{4\epsilon_{n_l}}{4\epsilon_{n_l}+1}}sin\ \omega_{n_l}t\right)|n_l\rangle|\phi_\downarrow\rangle \tag{6}$$

Therefore, we see how there is one oscillation between the spin-orbit states $|n_l-1\rangle|\phi_\uparrow\rangle$ and $|n_l\rangle|\phi_\downarrow\rangle$, in such a form that the change of spin polarization implies one for the orbital and vice versa. However, these abstracts does not take into account the topology of the system and are not enough to treat relativistic phonons into TIs. With this purpose we are going to introduce it into the adiabatic context to see explicitly how they affect to the topological properties and thus to the thermoelectric response in TIs.

Supplementary Note 2. The adiabatic Dirac oscillator

Coming back to the Eq. (1) we can rearrange its basis allowing its separation into two non-interacting and time-reversal counterparts $H_\pm(\boldsymbol{k} \mp e/\hbar\ \boldsymbol{a})$ in the same way as the 2D effective Hamiltonian used to describe the physics inside 2D and 3D TIs thin-films

$$H_\pm(\boldsymbol{k'}) = \begin{bmatrix} \pm m_e v_F^2 & \hbar v_F[(k_x \mp e/\hbar\ a_x) - i(k_y \mp e/\hbar\ a_y)] \\ \hbar v_F[(k_x \mp e/\hbar\ a_x) + i(k_y \mp e/\hbar\ a_y)] & \mp m_e v_F^2 \end{bmatrix} \tag{7}$$

where $\boldsymbol{a} = (-m\omega y/e, m\omega x/e, 0) = (-\mathcal{B}y/2, \mathcal{B}x/2, 0)$ is the vector potential which defines a magnetic field $\mathcal{B}$ of opposite sign for each branch of $H_\pm$. The form in which perturbation enters

guaranties time-reversal symmetry conservation and its spin-orbit nature manifested in its non-relativistic limit. Given that $H\pm$ are non-interacting we can work with one of the subsystems (we choose H_+) and then extend our results to the other. The unperturbed eigenstates of Eq. (7) are the eigenstates of the Dirac Hamiltonian H_{2D}, which for H_+ results

$$|n\rangle = \frac{1}{\sqrt{2}}\begin{bmatrix} \sqrt{1+\frac{M(\mathbf{k})}{\xi}} \\ e^{i\phi}\sqrt{1-\frac{M(\mathbf{k})}{\xi}} \end{bmatrix} \qquad |m\rangle = \frac{1}{\sqrt{2}}\begin{bmatrix} \sqrt{1-\frac{M(\mathbf{k})}{\xi}} \\ -e^{i\phi}\sqrt{1+\frac{M(\mathbf{k})}{\xi}} \end{bmatrix} \tag{8}$$

being $|n\rangle$ and $|m\rangle$ correspond to the states associated to the positive and negative energy solutions $\xi = \pm\sqrt{M^2(k)+\hbar^2 v_F^2 k^2}$. Generally speaking, $M(k) = M - Bk^2$, but this case will be discussed later given that our field $\boldsymbol{b}$ has been calculated without taking into account any dependence on the Hamiltonian parameter B. Thus, for the moment we are going to consider $M(k) = m_e v_F^2$. Now we are going to compute the correction to the eigenstates, that for an adiabatic perturbation and taking into account that $\xi_n - \xi_m = 2\xi$ results to be

$$|n\rangle \to |n\rangle - i\hbar\frac{\partial k_x}{\partial t}\frac{\langle m|\partial_{k_x} n\rangle}{2\xi}|m\rangle - i\hbar\frac{\partial k_y}{\partial t}\frac{\langle m|\partial_{k_y} n\rangle}{2\xi}|m\rangle \tag{9}$$

$$|m\rangle \to |m\rangle + i\hbar\frac{\partial k_x}{\partial t}\frac{\langle n|\partial_{k_x} m\rangle}{2\xi}|n\rangle + i\hbar\frac{\partial k_y}{\partial t}\frac{\langle n|\partial_{k_y} m\rangle}{2\xi}|n\rangle \tag{10}$$

It is the moment to introduce the perturbation given by the Dirac oscillator, which as we saw, enters into the form of a magnetic field for each branch. We have to be careful about the term $\partial k_i/\partial t$ because quantically the velocity $\hat{v}$ and the momentum operator $\hat{p}$ might present a different relationship to which present in a classical system. In relativistic quantum mechanics the velocity operator is defined as $\hat{v}_j = d\hat{x}_j/dt = \frac{i}{\hbar}[\hat{H},\hat{x}] = c\hat{\alpha}_j$, so that its introduction in the previous equations involves gauge dependent terms that made difficult to give an analytical expression to the corrections. This can be solved by noticing that we are introducing a Lorenz force in the system, in such a way that the correction to the momentum for a given direction must be proportional to the momentum in a perpendicular direction, that is why we take

$\partial k_i/\partial t = \epsilon_{ijk}k_j\omega_k$ in order to be consistent. Thus, we can proceed to rewrite the correction to the eigenstates

$$|n\rangle \to |n\rangle + \frac{\hbar\omega}{2\xi}\frac{\hbar v_F k}{2\xi}|m\rangle \qquad |m\rangle \to |m\rangle - \frac{\hbar\omega}{2\xi}\frac{\hbar v_F k}{2\xi}|n\rangle \tag{11}$$

and to compute analytically Berry curvature corrections $\Omega^n_{k_x,k_y} = i(\langle\partial_{k_x}n|\partial_{k_y}n\rangle - \langle\partial_{k_y}n|\partial_{k_x}n\rangle)$ after some algebra. Several cases will be analyzed which demonstrates the correctness of the topological intrinsic b that we have calculated.

Constant ω

The first case corresponds to a purely constant ω, i.e, the phonon frequency does not incorporate any dependence on the position r, the energy or the temperature. In that situation, the correction to the Berry curvature of the conduction band of H_+ results (Fig. S1)

$$\Omega^n_{k_x,k_y} \to \Omega^n_{k_x,k_y} - \frac{\hbar\omega}{2\xi}\frac{M}{\xi}\Omega^n_{k_x,k_y} + \frac{\hbar\omega}{2\xi}\frac{\hbar^2 v_F^2}{2\xi^2} - \frac{\hbar\omega}{2\xi}\frac{\hbar^2 v_F^2}{\xi^2}\left(1 - \frac{M^2}{\xi^2}\right) \tag{12}$$

The perturbation introduced produces a correction to the Berry curvature whose direction will depend on the sign of ω. It is straightforward to note that the corrections introduced preserves particle-hole symmetry $\hat{C}$ ($\xi \to -\xi$) as well as time-reversal $\hat{T}$ ($M \to -M, \omega \to -\omega$). At critical frequencies $\omega_c = eb/2m$, the magnitude of the perturbation has the value of the unperturbed Berry curvature at $k = 0$ evidencing the good interpretation of our approximation for the topological intrinsic field $b \approx 2M^2/\hbar e v_F^2$ as a measure of the robustness of the topological regime. The behaviour of the corrections is what one can expect from a constant perturbation into the real space and its homologous field into the k-space. This case might be enough to introduce certain phonons in topological insulators, however, it can not be applied generally given that thermal excitations and the majority of the phonons have an energy dependent nature.

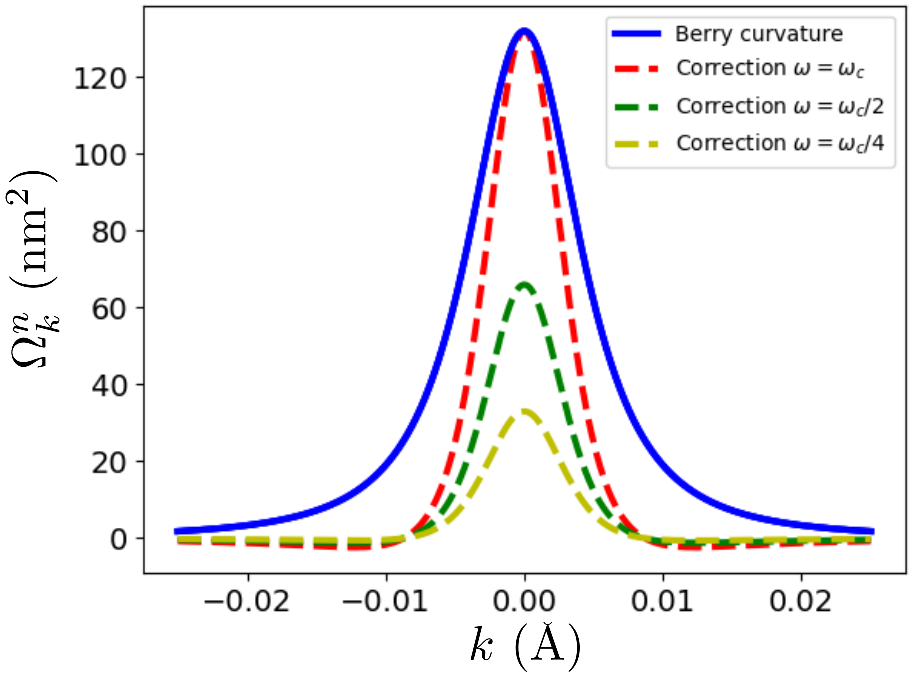

Figure S1. Unperturbed Berry curvature (blue solid line) of the conduction band of a Dirac Hamiltonian H_+ and first-order correction to it (dashed lines) for different constant frequencies below the critical value $\omega_c = eb/2m$. The parameters used are $M = -0.025$ eV, $B = 0$ and $v_F = 6.17\ 10^5$m/s.

Energy dependent ω

Given the physical equivalence between the frequency ω given by the Dirac oscillator Hamiltonian and the field $b = 2m\xi/\hbar e$ we are going to suppose the phonon frequency to have an energy dependence of the type $\omega = eb/2m = \xi/\hbar$.

$$|n\rangle \rightarrow |n\rangle + \frac{\lambda}{2}\frac{\hbar v_F k}{2\xi}|m\rangle \qquad\qquad |m\rangle \rightarrow |m\rangle - \frac{\lambda}{2}\frac{\hbar v_F k}{2\xi}|n\rangle \tag{13}$$

This allows us exploring the interpretation on the b field and to demonstrate that oscillation below this limit could modulate the Berry curvature by non producing changes into their topological properties. If our interpretation of the field b as a translation of the Berry curvature onto the real space is correct, then its corrections should result in a function of the type of the unperturbed Berry curvature. In this case, the correction to the Berry curvature is slightly different from the previous situation

$$\Omega^n_{k_x,k_y} \rightarrow \Omega^n_{k_x,k_y} + \lambda\frac{1}{2}\left(-\frac{\hbar^2 v_F^2}{2\xi^2} + \frac{\hbar^2 v_F^2}{2\xi^2}\frac{\hbar^2 v_F^2 k^2}{\xi^2} - \frac{M}{\xi}\Omega^n\right) = \Omega^n - \lambda\frac{M}{\xi}\Omega^n \tag{14}$$

where λ is a dimensionless parameter to measure the strength $[0, 1]$ of the perturbation, i.e, $\lambda = 1$ correspond to the case of a frequency $\omega = eb/2m$. The correction results in a simpler expression and which fits better to a function of the type of the Berry curvature. Although it is masked in the calculation, since we cancelled both ω and ξ, one can also check that the results also preserves $\hat{C}$ and $\hat{T}$ symmetries as in the previous case. With these ingredients, we are in a position to treat phonons and thermal oscillations into TIs. Despite of the conditions seem to be a bit restrictive, given that we are considering only in-plane oscillations, they are not unrealistic. It has been shown how polar optical modes can strongly couple with the topological electrons when the Fermi level lies close to the Dirac point as well as it has been reported how these modes present a linear dispersion law at low k that allow them to fulfill the conditions

underlined in the article [34].

Incorporating the Hamiltonian parameter B

One more case it is needed to complete the current analysis and it corresponds to incorporate the $-Bk^2$ term to the particles mass $M(k)$. This should be done to put the previous calculations closer to the context of TIs rather than in purely relativistic quantum mechanics in the vacuum. Obviously, in part, it has been already done once we considered the Fermi velocity v_F instead of the light velocity c in our formalism. Essentially, corrections remain equal with the substitution of M by $M - Bk^2$ but we have to add an additional term in the case of a constant ω. Thus, in the first case

$$\Omega^n_{k_x,k_y} \to \Omega^n_{k_x,k_y} - \frac{\hbar\omega}{2\xi}\frac{M(k)}{\xi}\Omega^n_{k_x,k_y} + \frac{\hbar\omega}{2\xi}\frac{\hbar^2 v_F^2}{2\xi^2} - \frac{\hbar\omega}{2\xi}\frac{\hbar^2 v_F^2}{\xi^2}\frac{\hbar^2 v_F^2 k^2}{\xi^2} + \frac{\hbar\omega}{2\xi}\frac{\hbar^2 v_F^2}{\xi^2}\frac{M(k)2Bk^2}{\xi^2} \tag{15}$$

where here clearly $\Omega^n = -\frac{\hbar^2 v_F^2 (M+Bk^2)}{2\xi^3}$ and $M(k) = M - Bk^2$. For an energy dependent ω

$$\Omega^n \to \Omega^n - \lambda\frac{(M - Bk^2)}{\xi}\Omega^n \tag{16}$$

Due to the addition of the $-Bk^2$ term corrections show to change their sign at a certain k in contrast with the Berry curvature. This is easily justified given that our approximation for b and thus for ω does not take into account any dependence on this term. Despite this, given that our approximation is valid for systems satisfying $v_F^2 >> 2MB/\hbar^2$, which is easily fulfilled thanks to the small gap and high Fermi velocity that typically characterizes 3DTI thin films, the addition of this term to the unperturbed Berry curvature does not produce any change of its sign in the mentioned limits where corrections tend to zero faster than Ω^n.

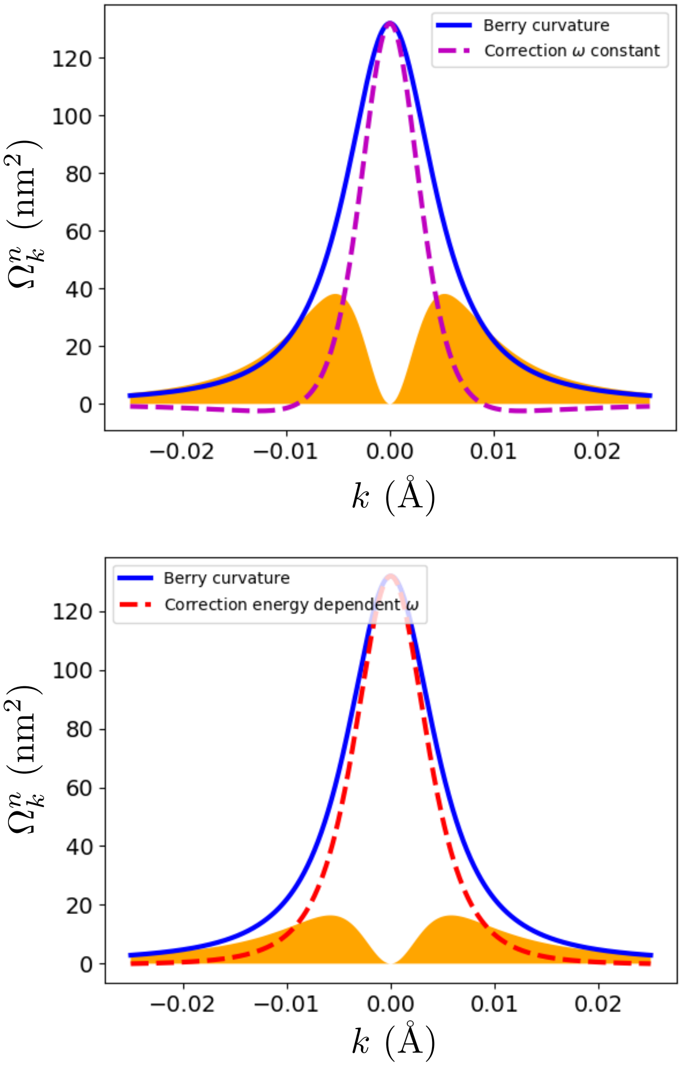

Figure S2. Unperturbed Berry curvature (blue solid line) of the conduction band of a non-trivial Dirac Hamiltonian H_+ $(M < 0, B < 0)$ and first-order correction to it (magenta and red dashed lines) for critical frequencies $\omega = eb/2m$ considering constant and energy dependences respectively. The parameters used are $M = -0.025$ eV, $B = -20$ eVÅ^2 and $v_F = 6.17\ 10^5$m/s. Filled regions correspond to the difference between the unperturbed Berry curvature and the first-order corrections showing no sign inversion even at critical frequencies.

References

[1] Xu, N., Xu, Y. & Zhu, J. Topological insulators for thermoelectrics. *npj Quantum Materials* **2**, 51 (2017).

[2] Venkatasubramanian, R., Siivola, E., Colpitts, T. & O'Quinn, B. Thin-film thermoelectric devices with high room-temperature figures of merit. *Nature* **413**, 597–602 (2001).

[3] Bernevig, B. A., Hughes, T. L. & Zhang, S.-C. Quantum spin Hall effect and topological phase transition in HgTe quantum wells. *Science* **314**, 1757–1761 (2006).

[4] Baldomir, D. & Faílde, D. On behind the physics of the thermoelectricity of topological insulators. *Scientific Reports* **9**, 6324 (2019).

[5] Liu, W., Jie, Q., Kim, H. S. & Ren, Z. Current progress and future challenges in thermoelectric power generation: From materials to devices. *Acta Materialia* **87**, 357 – 376 (2015).

[6] Nakahara, M. *Geometry, topology and physics* (CRC Press, 2003).

[7] Kane, C. L. & Mele, E. J. Z_2 topological order and the quantum spin Hall effect. *Phys. Rev. Lett.* **95**, 146802 (2005).

[8] Schnyder, A. P., Ryu, S., Furusaki, A. & Ludwig, A. W. W. Classification of topological insulators and superconductors in three spatial dimensions. *Phys. Rev. B* **78**, 195125 (2008).

[9] Roy, R. Topological phases and the quantum spin Hall effect in three dimensions. *Phys. Rev. B* **79**, 195322 (2009).

[10] Avron, J. E., Seiler, R. & Simon, B. Homotopy and quantization in condensed matter physics. *Phys. Rev. Lett.* **51**, 51–53 (1983).

[11] Kane, C. L. & Mele, E. J. Quantum spin Hall effect in graphene. *Phys. Rev. Lett.* **95**, 226801 (2005).

[12] Murakami, S. Quantum spin Hall effect and enhanced magnetic response by spin-orbit coupling. *Phys. Rev. Lett.* **97**, 236805 (2006).

[13] Sheng, D. N., Weng, Z. Y., Sheng, L. & Haldane, F. D. M. Quantum spin-Hall effect and topologically invariant Chern numbers. *Phys. Rev. Lett.* **97**, 036808 (2006).

[14] Hasan, M. Z. & Kane, C. L. Colloquium: Topological insulators. *Rev. Mod. Phys.* **82**, 3045–3067 (2010).

[15] Qi, X.-L., Hughes, T. L. & Zhang, S.-C. Topological field theory of time-reversal invariant insulators. *Phys. Rev. B* **78**, 195424 (2008).

[16] Wu, L. *et al.* Quantized Faraday and Kerr rotation and axion electrodynamics of a 3D topological insulator. *Science* **354**, 1124–1127 (2016).

[17] Essin, A. M., Moore, J. E. & Vanderbilt, D. Magnetoelectric polarizability and axion electrodynamics in crystalline insulators. *Phys. Rev. Lett.* **102**, 146805 (2009).

[18] Olsen, T., Taherinejad, M., Vanderbilt, D. & Souza, I. Surface theorem for the Chern-Simons axion coupling. *Phys. Rev. B* **95**, 075137 (2017).

[19] Moshinsky, M. & Szczepaniak, A. The Dirac oscillator. *Journal of Physics A: Mathematical and General* **22**, L817 (1989).

[20] Takahashi, R. & Murakami, S. Thermoelectric transport in perfectly conducting channels in quantum spin Hall systems. *Phys. Rev. B* **81**, 161302 2010).

[21] Zhou, B., Lu, H.-Z., Chu, R.-L., Shen, S.-Q. & Niu, Q. Finite size effects on helical edge states in a quantum spin-Hall system. *Phys. Rev. Lett.* **101**, 246807 (2008).

[22] Lu, H.-Z., Shan, W.-Y., Yao, W., Niu, Q. & Shen, S.-Q. Massive Dirac fermions and spin physics in an ultrathin film of topological insulator. *Phys. Rev. B* **81**, 115407 (2010).

[23] Zhao, L. *et al.* Singular robust room-temperature spin response from topological Dirac fermions. *Nature Materials* **13**, 580–585 (2014).

[24] Li, H., Sheng, L., Sheng, D. N. & Xing, D. Y. Chern number of thin films of the topological insulator Bi_2Se_3. *Phys. Rev. B* **82**, 165104 (2010).

[25] Bernevig, B. A. & Zhang, S.-C. Quantum spin Hall effect. *Phys. Rev. Lett.* **96**, 106802 (2006).

[26] König, M. *et al.* Quantum spin Hall insulator state in HgTe quantum wells. *Science* **318**, 766–770 (2007).

[27] Batra, I. P. From uncertainty to certainty in quantum conductance of nanowires. *Solid State Communications* **124**, 463–467 (2002).

[28] Dunne, G. V. & Schubert, C. Worldline instantons and pair production in inhomogenous fields. *Phys. Rev. D* **72**, 105004 (2005).

[29] Andrade, F. M. & Silva, E. O. Remarks on the Dirac oscillator in $(2 + 1)$ dimensions. *EPL (Europhysics Letters)* **108**, 30003 (2014).

[30] Bermudez, A., Martin-Delgado, M. A. & Solano, E. Exact mapping of the $2+1$ Dirac oscillator onto the Jaynes-Cummings model: Ion-trap experimental proposal. *Phys. Rev. A* **76**, 041801 (2007).

[31] Rozmej, P. & Arvieu, R. The Dirac oscillator. a relativistic version of the Jaynes-Cummings model. *Journal of Physics A: Mathematical and General* **32**, 5367–5382 (1999).

[32] Shen, S.-Q. *Topological insulators*, vol. 174 (Springer, Berlin, 2012).

[33] Vanderbilt, D. *Berry Phases in Electronic Structure Theory: Electric Polarization, Orbital Magnetization and Topological Insulators* (Cambridge University Press, 2018).

[34] Heid, R., Sklyadneva, I. Y. & Chulkov, E. V. Electron-phonon coupling in topological surface states: The role of polar optical modes. *Scientific Reports* **7**, 1095 (2017).

[35] Pan, H., Wu, M., Liu, Y. & Yang, S. A. Electric control of topological phase transitions in Dirac semimetal thin films. *Scientific Reports* **5**, 14639 (2015).

[36] Heisenberg, W. & Euler, H. Folgerungen aus der Diracschen Theorie des Positrons. *Zeitschrift für Physik* **98**, 714–732 (1936).

[37] Hastings, M. B. Topological order at nonzero temperature. *Phys. Rev. Lett.* **107**, 210501 (2011).

[38] Park, B. C. *et al.* Terahertz single conductance quantum and topological phase transitions in topological insulator Bi_2Se_3 ultrathin films. *Nature Communications* **6**, 6552 (2015).

[39] Yan, J., Zhang, Y., Kim, P. & Pinczuk, A. Electric field effect tuning of electron-phonon coupling in graphene. *Phys. Rev. Lett.* **98**, 166802 (2007).

[40] Tretiakov, O. A., Abanov, A., Murakami, S. & Sinova, J. Large thermoelectric figure of merit for three-dimensional topological anderson insulators via line dislocation engineering. *Applied Physics Letters* **97**, 073108 (2010).

[41] Chakraborty, P., Cao, L. & Wang, Y. Ultralow lattice thermal conductivity of the random multilayer structure with lattice imperfections. *Scientific Reports* **7**, 8134 (2017).
